# Albumin in Tears Modulates Bacterial Susceptibility to Topical Antibiotics in Ophthalmology

**DOI:** 10.3389/fmed.2021.663212

**Published:** 2021-11-30

**Authors:** Lionel Sebbag, Victoria L. Broadbent, Danielle E. Kenne, Ashtyn L. Perrin, Jonathan P. Mochel

**Affiliations:** ^1^Koret School of Veterinary Medicine, Hebrew University of Jerusalem, Rehovot, Israel; ^2^Department of Biomedical Sciences, SMART Pharmacology, College of Veterinary Medicine, Iowa State University, Ames, IA, United States; ^3^Veterinary Diagnostic Laboratory, College of Veterinary Medicine, Iowa State University, Ames, IA, United States

**Keywords:** antimicrobial susceptibility testing, antibiotic resistance, protein binding, bacterial keratitis, translational research, minimal inhibitory concentrations, tear fluid

## Abstract

Bacterial keratitis is a serious and vision-threatening condition in veterinary and human patients, one that often requires culture and susceptibility testing to adjust therapy and improve clinical outcomes. The present study challenges the antimicrobial susceptibility testing (AST) paradigm in ophthalmology, enabling more accurate *in vitro*-to-*in vivo* translation by incorporating factors normally present during host-pathogen interactions in clinical patients. Thirty bacteria (10 *Staphylococcus pseudintermedius*, 10 *Streptococcus canis*, 10 *Pseudomonas aeruginosa*) were isolated from canine patients with infectious keratitis. For each isolate, commercial plates (Sensititre™ JOEYE2) were used to assess the minimal inhibitory concentration (MIC) of 17 different antibiotics in the absence (0% albumin, control) or presence of canine albumin (0.01–2%). For *Staphylococcus pseudintermedius*, the experiment was repeated with actual tear fluid collected from canine eyes with ocular surface inflammation. Kruskal-Wallis, Wilcoxon signed rank test and Spearman's correlation tests were used for statistical analysis. Clinical outcomes were unfavorable in selected canine patients with bacterial keratitis (e.g., globe perforation, graft dehiscence) despite standard AST (i.e., 0% albumin in test medium) confirming that most corneal infections (93%) were susceptible to ≥1 topical antibiotics used at the initial visit. Albumin levels ≥0.05% increased MICs in a dose-dependent, bacteria-specific, and antibiotic-specific manner. No significant differences (*P* = 1.000) were noted in MICs of any antibiotic whether albumin or tear fluid was added to the Mueller-Hinton broth. Percent protein binding inherent to each antibiotic was associated with clinical interpretations (Spearman's rho = −0.53, *P* = 0.034) but not changes in MICs. Albumin in tears impacted the efficacy of selected ophthalmic antibiotics as only the unbound portion of an antibiotic is microbiologically active. The present findings could improve decision making of clinicians managing bacterial keratitis, reduce development of antimicrobial resistance, influence current guidelines set by the Clinical and Laboratory Standards Institute, and serve as a reference for bacteriological evaluations across medical fields and across species.

## Introduction

Antimicrobial susceptibility testing (AST) determines the lowest concentration of an antimicrobial agent that prevents visible growth of a microorganism using an agar or broth dilution susceptibility test. This information is used to define the isolate as either *susceptible, intermediate* or *resistant* based on clinical guidelines set by the Clinical and Laboratory Standards Institute (CLSI) (1). Although the AST bioassay has been immensely valuable in several medical fields since its standardization in 1961 (2), the testing pipeline is intrinsically flawed as the *in vitro* AST often fails to incorporate key environmental factors normally present during *in vivo* host-pathogen interactions. Consequently, differences between *in vitro* testing and *in vivo* clinical responses to antimicrobial agents are well-documented in the scientific literature (3–5), contributing in part to suboptimal clinical outcomes and the emergence of multi-drug resistance (6).

In the field of ophthalmology, discrepancies between testing conditions in *in vitro* AST vs. actual *in vivo* usage could explain why patients with infectious keratitis continue to deteriorate clinically (up to corneal perforation and vision loss) despite the use of an antibiotic shown to be effective *in vitro*. In particular, the ocular surface of diseased (inflamed) eyes contains high levels of albumin, a finding that is not accounted for in current AST guidelines. Indeed, ocular disease results in the breakdown of the blood-tear barrier—a phenomenon described in humans (7), dogs (8–10), and horses (11)—allowing for serum albumin to diffuse into the tear compartment through leaky conjunctival vessels. In turn, the presence of albumin in tears could impact the antimicrobial efficacy of a given antibiotic as only the unbound fraction of an antibiotic is known to be microbiologically active (12, 13).

Using a prospective study and standardized protocol, we aimed to (1) determine the impact of a wide range of albumin concentrations on the efficacy of 17 topical antibiotics against the most common ocular isolates identified in veterinary patients with bacterial keratitis; and (2) determine whether the impact of albumin is modulated by the presence of other key proteins (e.g., lysozyme, lactoferrin, and immunoglobulins) that compose the tear film in clinical patients. We hypothesized that albumin would reduce the sensitivity of ocular isolates to topical antibiotics, an effect that would be dose-dependent (0.01–2%), antibiotic-dependent (17 antibiotics tested), and bacteria-dependent (30 bacterial isolates tested). Further, we hypothesized that the changes in AST observed with albumin would not differ from the changes observed with tear fluid collected from patients, confirming that albumin alone could be used by diagnostic laboratories to optimize the clinical usefulness of this important bioassay.

## Materials and Methods

### Ocular Isolates

The study focused on the 3 most common bacterial species identified in dogs with ulcerative keratitis, that is, *Staphylococcus pseudintermedius, Streptococcus canis*, and *Pseudomonas aeruginosa* (6, 14). Ten bacterial isolates were selected for each bacterial species, providing a total of 30 bacterial isolates for the study. The selection was based on the availability of a given bacterial isolate in the −80°C freezer of the microbiology laboratory (2015–2020), as well as availability of medical information regarding the history and clinical outcome of the canine patient from whom the isolate was retrieved.

### Albumin Solutions

Canine albumin powder (Animal Blood Resources International, Stockbridge, MI) was mixed with phosphate-buffered saline (PBS 1X, Gibco™, Thermo Fisher Scientific Inc., Waltham, WA) in a sterile manner under a laminar flow hood by a pharmacist, formulating 10 albumin solutions including 0% (control), 0.02, 0.1, 0.2, 0.4, 0.7, 1, 1.5, 2, and 4%. Accounting for a 2-fold dilution from mixing 1:1 with bacterial broth (see section Antimicrobial Susceptibility Testing), these 10 solutions provided albumin concentrations (0, 0.01, 0.05, 0.1, 0.2, 0.35, 0.5, 0.75, and 1, 2%) that represent a spectrum of albumin detected in tears of healthy canine eyes (generally ≤0.1%) and canine eyes with mild to severe conjunctival inflammation (generally 0.1–2%). This information was based on two previous reports (8, 10) as well as prospective evaluation of 25 canine patients diagnosed with ulcerative keratitis at the Iowa State University's Lloyd Veterinary Medical Center, collecting and analyzing tear samples for albumin content as previously described (10). Briefly, tear collection was performed with dye-free Schirmer strips (Eye Care Product Manufacturing, Tucson, AZ) wetted until 20-mm mark, followed by centrifugation in pre-punctured 0.2-mL tubes at 3,884 g for 2 min and albumin quantification with a canine-specific ELISA kit (Serum albumin ELISA kit, Life Span Biosciences, Inc., Seattle, WA) following the manufacturer's guidelines.

### Tear Collection From Diseased Canine Eyes

Ten Beagle dogs (5 male neutered, 5 female spayed) were recruited for this experiment, aged 30.8 ± 0.8 months (30–32 months) and weighing 9.9 ± 0.7 kg (8.7–11.3 kg), all confirmed to be ophthalmoscopically healthy based on slit lamp biomicroscopy (SL-17; Kowa Company, Ltd., Tokyo, Japan), indirect ophthalmoscopy (Keeler Vantage; Keeler Instruments, Inc., Broomall, PA, USA), rebound tonometry (TonoVet; Icare Finland Oy, Espoo, Finland), and Schirmer tear test-1 (STT-1; Eye Care Product Manufacturing LLC, Tucson, AZ).

A large volume of tears (18 mL) was collected from dogs with ocular surface inflammation to provide biological fluid for the subsequent *in vitro* experiments. First, conjunctivitis was induced in both eyes with topical administration of 1% histamine solution, as previously described (8, 10). Twenty minutes were allowed to pass for conjunctivitis to fully develop and ocular surface homeostasis to be restored (8, 15). Then, tear collection was performed in all dogs by placing 4 × 10 mm strips of polyvinyl acetal sponges in the ventral conjunctival fornix as previously described (16, 17). A total of 15 tear collection sessions were conducted in each canine eye over a 2-day period, ensuring a minimum of 10 min between tear collections (9, 18) and maintaining conjunctival inflammation with repeated topical histamine administration when conjunctival swelling subsided. All animal experiments (sections Albumin Solutions and Tear Collection From Diseased Canine Eyes) were approved by the Institutional Animal Care and Use Committee at Iowa State University (protocol # 19-360).

### Antimicrobial Susceptibility Testing

#### Albumin Solutions

Bacterial isolates (*n* = 30) cultured from canine patients were revived from the −80 ± 10°C freezer by thawing at room temperature and grown on tryptic soy with 5% sheep blood agar plates. All plates were then incubated at 35 ± 2°C with 5–10% CO_2_ for a total length of 24–48 h. Using the Sensititre 0.5 McFarland Standard (catalog # E1041, Thermo Scientific Inc.), the Sensititre nephelometer (Thermo Scientific Inc.) was calibrated prior to making each bacterial suspension in 0.85% physiological sterile saline to a known dilution of 10^7^ colony forming units per milliliter (CFU/mL). Twenty microliter of each bacterial suspension was then transferred to standard Mueller-Hinton broth (MHB; catalog #T3462, Thermo Scientific Inc.) for *Staphylococcus pseudintermedius* and *Pseudomonas aeruginosa*; or MHB with lysed horse blood (catalog # CP114-10, Thermo Scientific Inc.) for *Streptococcus canis*. To ensure the inoculation of the bacterial suspension was pure, 1 mL of solution was plated onto a 5% Sheep Blood agar plate, streaked for isolation, and incubated at 35 ± 2°C for 16–24 h. Once confirmed as a pure culture, susceptibility plates were read.

For each bacterial isolate, two broths (2 × 11 mL) were prepared and aliquoted into two sets of tubes: (i) 0.25 mL transferred to ten 2-mL Eppendorf tubes containing equal volume of albumin solution, one tube per albumin concentration (0–4%); and (ii) 1.6 mL transferred to ten 5-mL glass tube containing equal volume of albumin solution, one tube per albumin concentration (0–4%). The first *set* served as positive control, while the second set served as study samples. Of note, the mixing of solutions resulted in 1:1 dilution of the bacterial broth with the albumin solution, hence the initial 2-fold higher concentration for the bacterial concentration in the broth and for the albumin concentration in each albumin solution.

Following gentle mixing of each tube for 30 s, solutions were transferred to test plates as follows (Supplementary Figure 1):

**(1) Positive control plate**: One blank plate with no antibiotics (Corning 96-well Clear Polystyrene Microplates, Corning Inc.) was used for each bacterial isolate, manually pipetting 50-μl of each solution (broth-albumin solution) into the 8 wells of each column (column 1 for albumin 0%, column 2 for albumin 0.01%, …, column 10 for albumin 2%).
**(2) Antimicrobial susceptibility plates**: An automated inoculation delivery system (Sensititre AIM™, Thermo Scientific Inc.) was used to transfer each broth-albumin solution onto standard plates (50-μL in each of the 48 wells) that are specifically manufactured for sensitivity testing in ophthalmology (JOEYE2 plate, Thermo Scientific Inc.). These plates are preloaded with serial dilutions of 17 different antibiotics that are commonly used in veterinary patients to manage bacterial keratitis, including erythromycin, oxytetracycline, gentamicin, chloramphenicol and ofloxacin (assets.thermofisher.com/TFS-Assets/MBD/Specification-Sheets/Sensititre-Plate-Layout-JOEYE2.pdf). A total of 5 JOEYE2 plates were used for each bacterial isolate since each plate can assess two separate sets of samples: plate 1 for albumin 0% (left half) and albumin 0.01% (right half),…, plate 5 for albumin 1% (left half) and albumin 2% (right half).

Following CLSI guidelines, all Sensititre plates were incubated at 37 ± 2°C for either 16–20 h (*Pseudomonas* sp.), 20–24 h (*Streptococcus* sp.), or 24 h (*Staphylococcus* sp.), followed by data recording using a digital MIC viewing system (Sensititre Vizion™, Thermo Scientific Inc.). The presence or absence of bacterial growth was recorded in each well of the blank plates. For each of the 17 antibiotics of JOEYE plates, the MIC was read as the lowest concentration of antimicrobial agent that completely inhibited organism growth, a result that was accompanied by a clinical interpretation (i.e., susceptible, intermediate, resistant, or non-interpretable) based on the breakpoints described in the VET08 and M100 CLSI documents (1, 19).

#### Tear Fluid

Similar experiments were conducted with canine tear fluid instead of albumin solutions, albeit with few modifications due to the relatively limited volume of tears available (18 mL). In particular, a single bacterial species (*Staphylococcus pseudintermedius, n* = 10 isolates) and a single albumin concentration (~0.5 mg/mL) were evaluated herein. First, albumin quantification with ELISA showed that median (mean) albumin concentration in the collected tear fluid was 1,002 μg/mL (1,010 μg/mL). Then, a Mueller-Hinton broth was prepared for each bacterial isolate as previously described, followed by mixing of equal volume (1.6 mL) of MHB and tear fluid in 5-mL glass tube (resulting in two-fold dilution of albumin levels in the tear fluid), and automated inoculation of the resulting broth/tears solutions onto JOEYE2 Sensititre plates. For positive controls, 50-μL of each broth/tears solution was pipetted into the 8-wells of each column from a blank plate with no antibiotics. In both cases, MICs were read following incubation at 37 ± 2°C for 24 h.

### Data Analysis

Normality of data was assessed using the Shapiro-Wilk test. For each antibiotic (*n* = 17) and bacterial species (*Staphylococcus pseudintermedius, Streptococcus canis, Pseudomonas aeruginosa*), MICs obtained with albumin (0.01–2%) were compared to control (albumin 0%) using the Kruskal-Wallis test and *post-hoc* Dunnett's method. The Wilcoxon signed rank test was used to compare MICs of each antibiotic for *Staphylococcus pseudintermedius* when the test medium was supplemented with albumin vs. tear fluid containing the same level of albumin. Last, Spearman's correlation tests were used to assess the association between percent protein binding (inherent to each antibiotic) and percent bacterial isolates that experienced changes in MICs or changes in clinical interpretations; of note, percent protein binding was derived from data in humans (and not dogs) due to lack of comprehensive characterization of protein binding for most antibiotics tested herein in the canine species. Statistical analyses were performed with SigmaPlot 14.0 (Systat software, Point Richmond, CA), and *P*-values < 0.05 were considered significant.

## Results

### Antimicrobial Susceptibility Testing

**Positive control**—Positive bacterial growth was noted in all 80 wells (8 × 10 levels of albumin) for each of the 30 bacterial isolates, indicating that albumin did not have antimicrobial properties on its own regardless of the protein concentration (0.01–2%). A representative example is depicted for an isolate of *Pseudomonas aeruginosa* in Supplementary Figure 2.

**Figure 1:**
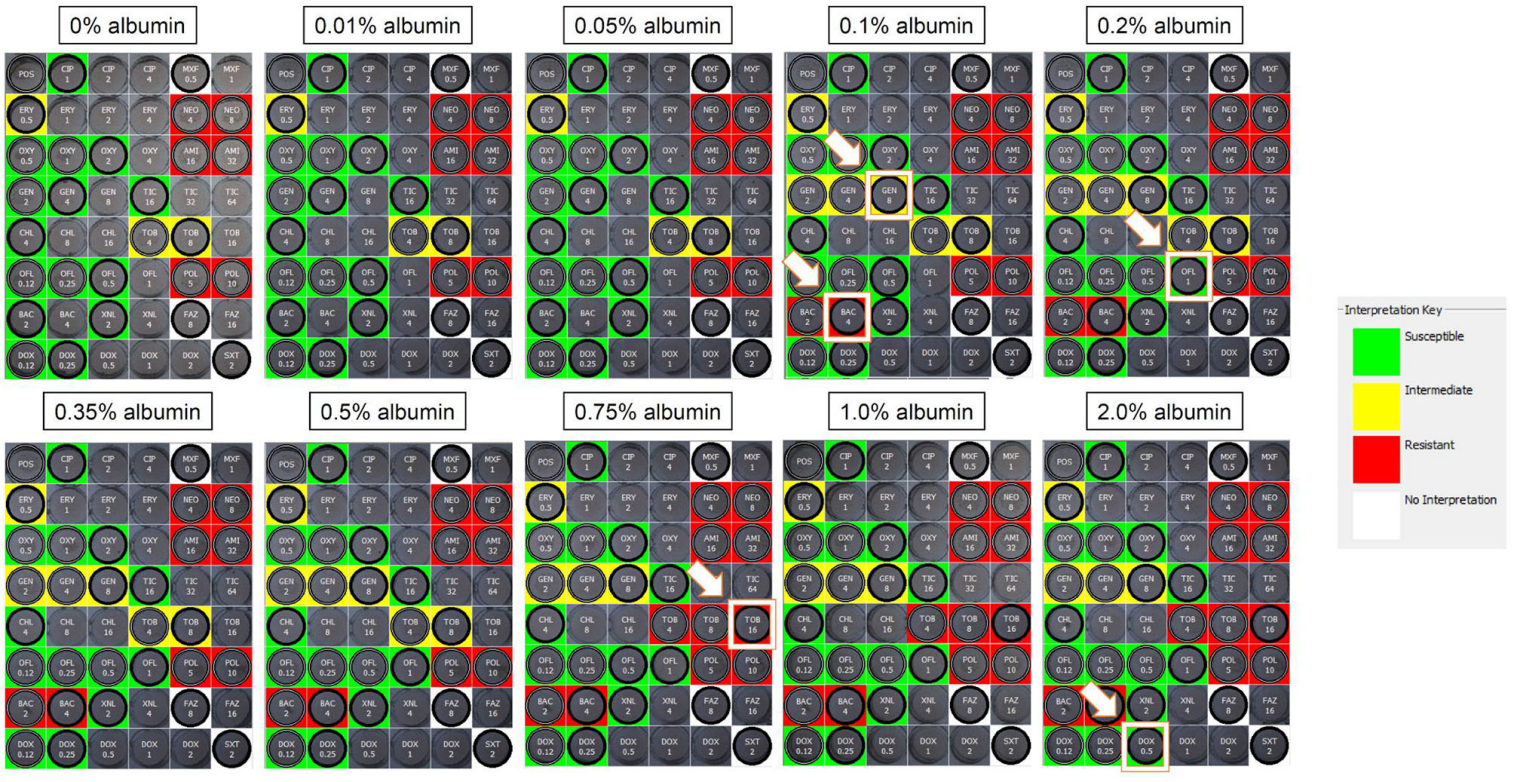
Sensititre™ JOEYE2 plates assessing the MICs of ophthalmic antibiotics against *Streptococcus canis* in the absence (0%) or presence of albumin (0.01–2%). Wells highlighted by a white border and white arrow represent an increased MIC from the previous albumin level. In this example, MICs increased by 2-fold for gentamicin (4–8 μg/ml), bacitracin (2–4 μg/ml), ofloxacin (0.5–1 μg/ml), tobramycin (8–16 μg/ml), and doxycycline (0.25–0.5 μg/ml), shifting the clinical interpretations from susceptible to resistant for bacitracin, susceptible to intermediate for gentamicin, and intermediate to resistant for tobramycin. MIC, minimal inhibitory concentration; Green, susceptible; Yellow, intermediate; Red, resistant; White, non-interpretable.

**Albumin solutions**—The impact of albumin on MIC was dependent on the protein concentration, the antibiotic, and the bacterial isolate. A representative example is depicted for an isolate of *Streptococcus canis* in Figure 1. In this example, changes in MICs were 2-fold increase for gentamicin (4–8 μg/mL), bacitracin (2–4 μg/mL), ofloxacin (0.5–1 μg/mL), tobramycin (8–16 μg/mL), and doxycycline (0.25–0.5 μg/mL). Such changes shifted the clinical interpretations from *susceptible* to *resistant* for bacitracin, *susceptible* to *intermediate* for gentamicin, and *intermediate* to *resistant* for tobramycin.

The median MIC did not change for any albumin concentration and any bacterial species for the following 7/17 antibiotics: amikacin, cefazolin, ceftiofur, ciprofloxacin, moxifloxacin, polymyxin B, and trimethoprim/sulfamethoxazole. For the other 10 antibiotics evaluated in this study, albumin levels ≥ 0.05% increased MICs in a dose-dependent, bacteria-specific and antibiotic-specific manner (Table 1). Overall, the antibiotics most affected by albumin were erythromycin (8-fold increased MIC_50_) and tobramycin (4-fold increased MIC_90_) for *Staphylococcus pseudintermedius*, doxycycline (4.2-fold increased MIC_50_) for *Streptococcus canis*, and ticarcillin (4-fold increased MIC_50_) for *Pseudomonas aeruginosa*. Of note, assessment of AST obtained with 0% albumin (i.e., 50% MHB) vs. 100% MHB (standard microbiology testing) showed 2–4-fold differences in MICs in 22/510 (4.3%) pairwise comparisons.

**Table 1:** Changes in MICs of ophthalmic antibiotics used against common bacterial species isolated in dogs with infectious keratitis.

	***Pseudomonas aeruginosa***									***Staphylococcus pseudintermedius***									***Streptococcus canis***								
**Albumin (mg/ml)**	**0.1**	**0.5**	**1**	**2**	**3.5**	**5**	**7.5**	**10**	**20**	**0.1**	**0.5**	**1**	**2**	**3.5**	**5**	**7.5**	**10**	**20**	**0.1**	**0.5**	**1**	**2**	**3.5**	**5**	**7.5**	**10**	**20**
**Antibiotic**																											
Bacitracin	–	–	–	–	–	–	–	–	–	–	–	–	–	–	–	–	–	–	–	–	1.5	2	2	2	2	2	2
Chloramphenicol	–	–	–	–	–	–	–	–	–	–	–	–	1.5	1.5	2	2	2	3	–	–	–	–	–	–	–	–	–
Doxycycline	–	–	–	–	–	–	–	–	–	–	–	–	1.5	2	2	2	2	2	–	1.5	1.5	1.5	2	2	2	3	3
Erythromycin	–	–	–	–	–	–	–	–	–	–	–	–	–	1.5	1.5	2	2	8	–	–	–	–	–	–	–	–	–
Gentamicin	–	–	–	–	–	–	–	–	–	–	–	–	–	1.5	2	2	2	2	–	–	–	–	–	2	2	2	2
Neomycin	–	–	2	2	2	2	2	2	2	–	–	–	–	–	–	–	–	–	–	–	–	–	–	–	–	–	–
Ofloxacin	–	–	–	1.5	2	2	2	2	2	–	–	–	–	1.5	1.5	2	2	3	–	–	–	2	2	2	2	2	2
Oxytetracycline	–	–	1.5	2	2	2	2	2	2	–	–	–	–	–	–	–	–	–	–	–	–	–	–	–	–	–	–
Ticarcillin	–	–	–	2	2	2	2	3	4	–	–	–	–	–	–	–	–	–	–	–	–	–	–	–	–	–	–
Tobramycin	–	–	–	–	–	–	–	–	–	–	–	–	–	–	–	–	–	–	–	–	–	–	–	2	2	2	2

*Results are depicted as fold increase in median MIC (10 isolates for each bacterial species) when comparing standard test medium (Mueller Hinton broth, no albumin) vs. Mueller Hinton broth supplemented with clinically relevant levels of canine albumin (0.1 to 20 mg/ml). – indicates no change in MIC. Boxes highlighted in gray indicate statistical significance (P < 0.05) compared to baseline susceptibility testing (0% albumin). MIC, minimal inhibitory concentration*.

Table 2 describes the percent of bacterial isolates affected by the addition of albumin in the AST medium, highlighting changes in MICs and clinical interpretations. Results varied among antibiotics and among bacterial species. For instance, albumin increased the MIC of ofloxacin in 100, 80, and 80% of *Pseudomonas aeruginosa, Staphylococcus pseudintermedius*, and *Streptococcus canis* isolates, respectively. The percent of protein binding inherent to each antibiotic (e.g., 32% for ofloxacin, 80–93% for erythromycin; Table 2) was not significantly correlated with the percent bacterial isolates with changes in MIC values (Spearman's rho = −0.31, *P* = 0.233), although a moderate and significant negative correlation was noted for changes in clinical interpretations (Spearman's rho = −0.53, *P* = 0.034).

**Table 2:** Frequency of bacterial isolates with (increased MIC)/(changes in clinical interpretation) when comparing standard test medium (Mueller Hinton broth, no albumin) vs. Mueller Hinton broth supplemented with clinically relevant levels of canine albumin.

		**Percent bacterial isolates with increased MIC/changes in clinical interpretation**			
		**from albumin added to the test medium**			
**Antibiotic**	**Protein binding (%)**	***Pseudomonas aeruginosa***	***Staphylococcus pseudintermedius***	***Streptococcus canis***	**All bacterial isolates**
Amikacin	≤10%	0/0	0/0	0/0	0/0
Bacitracin	Not available	0/0	0/0	60/60	20/20
Cefazolin	74–86%	0/0	0/0	0/0	0/0
Ceftiofur	>90%	0/0	0/0	0/0	0/0
Chloramphenicol	50–60%	0/0	100/50	0/0	33/17
Ciprofloxacin	20–40%	0/0	0/0	10/10	3/3
Doxycycline	>90%	0/0	60/0	70/0	43/0
Erythromycin	80–93%	0/0	40/30	0/0	13/10
Gentamicin	0–30%	0/0	90/30	100/100	63/43
Moxifloxacin	50%	20/0	10/0	0/0	10/0
Neomycin	40%	70/0	10/0	0/0	27/0
Ofloxacin	32%	100/90	80/30	80/70	87/63
Oxytetracycline	20–25%	70/80	10/10	10/0	30/30
Polymyxin B	92–99%	10/0	0/0	0/0	3/0
Ticarcillin	45%	100/90	10/10	0/0	37/33
Tobramycin	<30%	0/0	30/30	100/100	43/43
Trimethoprim/sulfamethoxazole	45–75%	0/0	0/0	0/0	0/0

*Percent protein binding inherent to each antibiotic is depicted in the second column for reference. MIC, minimal inhibitory concentration*.

**Tear fluid**—Positive bacterial growth was noted in all 80 wells (8 × 10 bacterial isolates), indicating that tear fluid did not have notable antimicrobial properties on its own. Further, no significant differences (*P* = 1.000) were noted in the median MIC of any antibiotic whether albumin or tear fluid was mixed to the Mueller Hinton broth. In fact, only 7 out of 170 (4.1%) pairwise comparisons had a different MIC with tear fluid vs. albumin.

### Clinical Outcomes of Patients With Bacterial Keratitis

**Figure 2:**
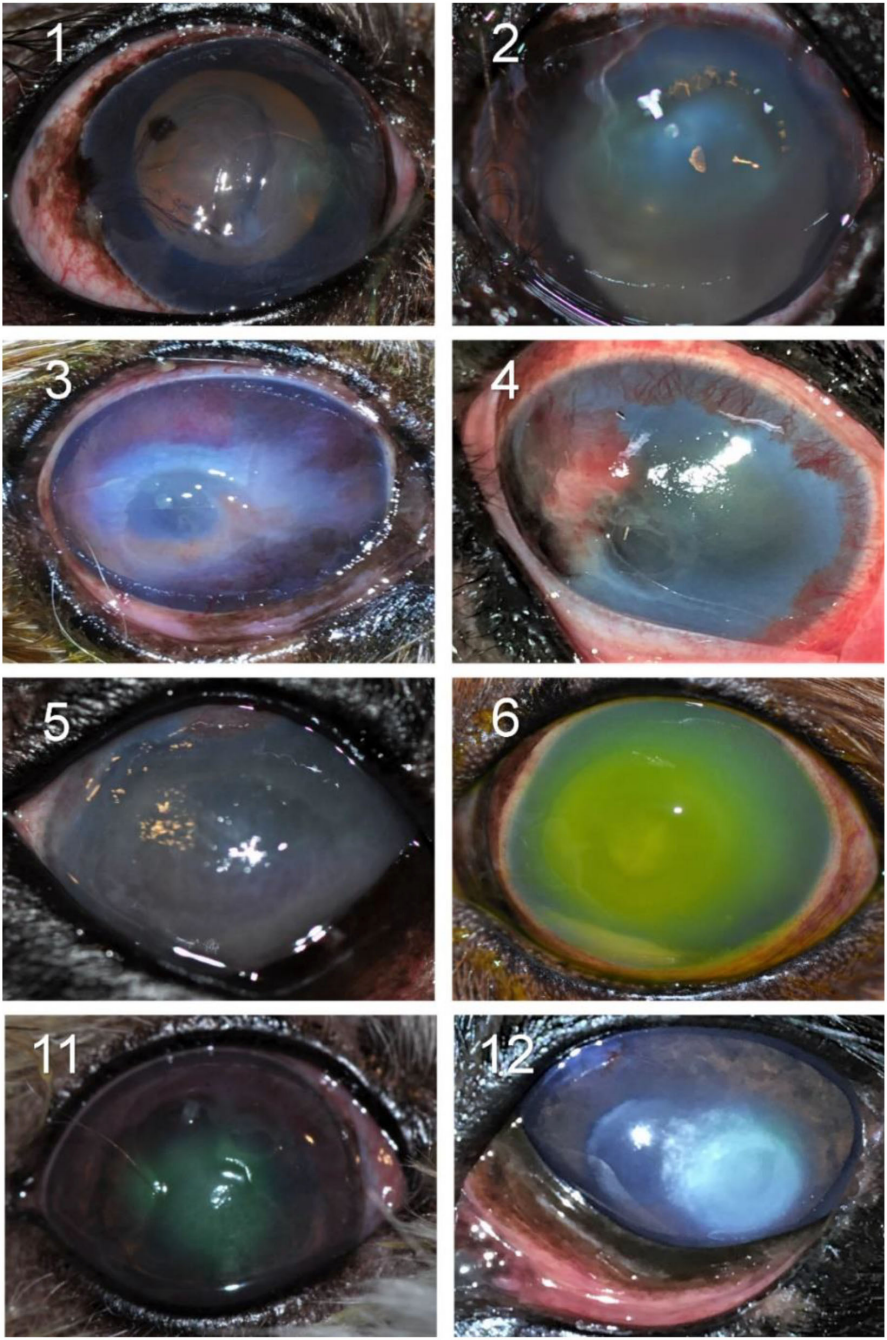
Clinical photographs of canine eyes diagnosed with culture-confirmed bacterial keratitis. Patients ID are shown in the top left (see Table 1 for additional details).

Details of the 30 canine patients with bacterial keratitis are described in Table 3, with representative clinical images depicted in Figure 2.

**Table 3:** Clinical description of 30 canine patients diagnosed with culture-confirmed bacterial keratitis and managed with medical ± surgical therapies.

**Bacterial isolate**	**Patient ID**	**Signalment**	**Concurrent systemic or ocular disease**	**Ulcer characteristics**	**Topical antibiotics (clinical interpretation 0% albumin/0.1% albumin)**	**Follow up**
*Pseudomonas aeruginosa*	1	11 yo FS Shih Tzu	Systemic hypertension	3 × 3 mm, 20% depth	Chloramphenicol **(R/R)** and Ofloxacin **(S/S)** 12× daily	Healed by D19
	2	11 yo MC Shih Tzu	Pseudophakia, ocular hypertension	5 × 5 mm, 10% depth	Chloramphenicol **(R/R)** and Ofloxacin **(S/I)** 8× daily	Initial worsening (50% depth), healed by D28 after adding gentamicin **(S/S)** 8× daily
	3	13 yo FS Cavalier King Charles Spaniel	None	5 × 5 mm, 30% depth	Chloramphenicol **(R/R)** 6× daily	Initial worsening (7 mm, focal perforation), healed by D50 after adding gentamicin **(S/S)** 10× daily
	4	12 yo FS English Spaniel	Hypothyroidism, systemic hypertension	4 × 4 mm, 50% depth	Chloramphenicol **(R/R)** and Ofloxacin **(S/I)** 6× daily	Initial worsening (80% depth), healed by D25 after conjunctival pedicle flap and adding Amikacin **(S/S)** 6× daily on D3
	5	12 FS Labrador retriever	Diabetes mellitus, systemic hypertension	10 × 6 mm, 20% depth	Chloramphenicol **(R/R)** and Ofloxacin **(S/S)** 12× daily	Healed by D14
	6	7 yo FS Cavalier King Charles Spaniel	Corneal dystrophy	10 × 8 mm, 70% depth	Tobramycin **(S/S)** and Ofloxacin **(S/S)** 8× daily	Initial improvement (D8) but acute worsening (focal perforation) on D15 and subsequent enucleation
	7	5 yo MC Shih Tzu	Atopic dermatitis, distichiasis	5 × 3 mm, perforation with fibrin	Biosynthetic corneal graft, Cefazolin **(R/R)** and Ofloxacin **(S/I)** 6× daily	Graft dehiscence D6, Conjunctival pedicle flap D11 (dehisced D18), healed with continued antibiotherapy [gentamicin **(S/S)**] by D120
	8	10 yo MC Shih Tzu	Atopic dermatitis, trichiasis	10 × 8 mm (50% depth) with 4 mm area of perforation	Conjunctival pedicle flap, Cefazolin **(R/R)** and Gentamicin **(S/S)** 6× daily	Ongoing keratomalacia and focal flap dehiscence D7, added amikacin **(S/S)** and ofloxacin **(S/S)**, healed by D14
	9	6 yo FS Havenese	None	8 × 4 mm (70% depth) with 3 mm area of perforation	Biosynthetic corneal graft, Tobramycin **(S/S)** and Moxifloxacin **(NI/NI)** 8× daily	Graft fully incorporated D17, healed well
	10	9 yo MC Shih Tzu	Keratoconjunctivitis sicca	10 × 8 mm, 80% depth	Cefazolin **(R/R)** and Ofloxacin **(I/I)** 12× daily	Progression to descemetocele by D14 (required conjunctival pedicle flap, healed by D39) despite switching to gentamicin **(S/S)** on D7 when culture results were back
*Staphylococcus pseudintermedius*	11	10 yo MC Shih Tzu	Neurogenic keratoconjunctivitis sicca	7 × 5 mm, 40% depth	Chloramphenicol **(S/S)** and Ofloxacin **(S/S)** 8× daily	Improvement noted on D3 and D9, fully healed by D30
	12	9 yo MC Longhaired Dachsund	Diabetes mellitus, Cushing's disease, pseudophakia	5 × 5 mm, 50% depth	Chloramphenicol **(S/S)** and Ofloxacin **(S/S)** 6× daily	Improvement noted on D5 and D10, fully healed by D19
	13	10 yo FS Rat terrier	Posterior lens luxation, glaucoma	7 × 5 mm, 20% depth	Chloramphenicol **(S/S)** and Moxifloxacin **(S/S)** 8× daily	Appears stable D4 and D10, sudden worsening D18 (8 mm size, keratomalacia), eye enucleated
	14	3 yo MC Shih Tzu	None	5 × 2 mm descemetocele	Conjunctival pedicle flap, Chloramphenicol **(S/S)** and Ofloxacin **(S/S)** 5× daily	Improved on D9, fully healed by D23
	15	16 yo MC Shih Tzu	Corneal degeneration, trichiasis	2 × 2 mm perforation	Cefazolin **(NI/NI)** and Ofloxacin **(S/S)** 8× daily	Stable on D3 (no re-perforation), healed by D24
	16	7 yo MC Boston terrier	Aphakia, glaucoma	8 × 6 mm, 50% depth	Cefazolin **(NI/NI)** and Tobramycin **(S/S)** 6× daily	Appeared stable on D7 but progressed to 90% depth on D17, eye enucleated
	17	9 yo MC Shih Tzu	Keratoconjunctivitis sicca	10 × 8 mm, 80% depth	Cefazolin **(NI/NI)** and Ofloxacin **(S/I)** 8× daily	Appeared stable on D7 but progressed to descemetocele D14, conjunctival pedicle flap + switched cefazolin to chloramphenicol **(S/S)**, healed by D39
	18	10 yo FS Miniature Pinscher	Diabetes mellitus, keratoconjunctivitis sicca	3 × 3 mm, superficial defect with stromal infiltrates	Chloramphenicol **(S/S)** and Tobramycin **(S/S)** 6× daily	Improved on D3, healed by D10
	19	6 yo FS Pomeranian	Neurotrophic keratopathy	7 × 4 mm, 60% depth	Chloramphenicol **(S/S)** and Tobramycin **(S/S)** 6× daily	Improved on D5 and D10, healed by D26
	20	12 yo FS Shih Tzu	None	4 × 3 mm, 30% depth peripherally and 70% depth centrally	Chloramphenicol **(S/S)** and Ofloxacin **(S/S)** 6× daily	Improved on D9, healed by D20
*Streptococcus canis*	21	8 yo MC Shih Tzu	None	2 × 2 mm descemetocele	Conjunctival pedicle flap, Cefazolin **(NI/NI)** and Ofloxacin **(S/I)** 6× daily	Focal suture dehiscence and graft retraction D11 (Seidel positive) and D18 (Seidel negative), healed by D32
	22	6 yo MC Pug	None	4 × 3 mm, 60% depth	Chloramphenicol **(S/S)** and Ofloxacin **(NI/NI)** 6× daily	Healed on D14
	23	10 MC Shih Tzu	None	3 × 3 mm, superficial defect with stromal infiltrates	Cefazolin **(NI/NI)** and Ofloxacin **(S/S)** 6× daily	Improved D3, healed D10
	24	9 yo MC Longhaired Dachsund	Diabetes mellitus, cataract	2 × 2 mm, 70% depth	Chloramphenicol **(S/S)** and Gentamicin **(S/S)** 12× daily	Improved D3 (mostly re-epithelialized) and D10, healed by D17
	25	11 yo MC Shih Tzu	Keratoconjunctivitis sicca, cataract, eyelid notch defect	7 × 5 mm, superficial defect	Chloramphenicol **(S/S)** and Gentamicin **(S/S)** 6× daily	Healed by D21 (first recheck)
	26	3 yo MC Shih Tzu	None	3 × 3 mm, 40% depth	Cefazolin **(NI/NI)** and Ofloxacin **(S/S)** 10× daily	Healed by D9 (first recheck)
	27	9 yo MC Boston terrier	Cushing's disease, infectious crystalline keratitis	8 × 4 mm, 50% depth	Cefazolin **(NI/NI)** and Gentamicin **(S/R)** 12× daily	Slightly deeper D5, added ciprofloxacin **(S/S)** but focal perforation (2 mm area) on D8, biosynthetic graft + conjunctival pedicle flap, healed by D33
	28	2 yo MC Pug	Entropion, distichiasis	3 × 4 mm, 30% depth	Chloramphenicol **(S/S)** and Tobramycin **(I/R)** 8× daily	Improved on D3, healed by D21
	29	8 yo MC Shih Tzu	Atopic dermatitis	4 × 3 mm descemetocele	Conjunctival pedicle flap, Chloramphenicol **(S/S)** and Ofloxacin **(S/S)** 6× daily	Improved on D7, healed by D21
	30	7 yo MC West Highland White terrier	Keratconjunctivitis sicca	3 × 3 mm, 30% depth	Cefazolin **(NI/NI)** and Ofloxacin **(S/S)** 6× daily	Healed by D7

*Bolded letters represent the clinical interpretations received by the clinician (letter before slash, issued from MIC testing with standard test medium) and the one extrapolated from MIC results for test medium supplemented with 0.1% albumin (letter after slash). S, Susceptible; I, Intermediate; R, Resistant; NI, Non-interpretable*.

Two out of 30 canine patients (6.7%) had bacterial isolates considered *intermediate* or *resistant* to the topical antibiotic(s) used at the initial visit; in these two cases, the corneal ulcer worsened and required the addition of another antibiotic (cases #3 and #10) as well as surgical stabilization with a conjunctival pedicle flap (case #10).

**Figure 3:**
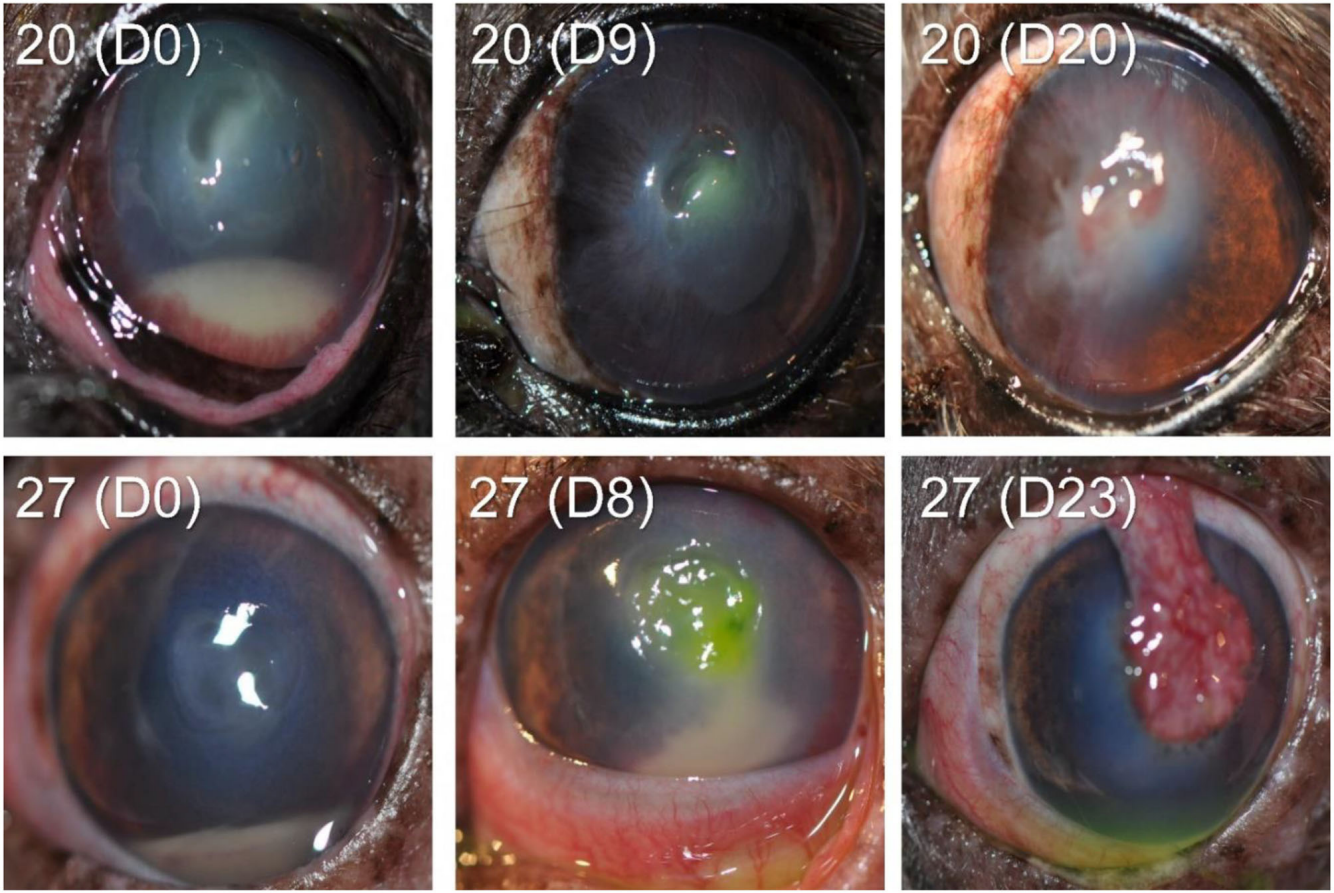
Clinical photographs at diagnosis and follow-up visits of canine eyes diagnosed with culture-confirmed bacterial keratitis. Patients ID are shown in the top left (see Table 1 for additional details).

**Figure 4:**
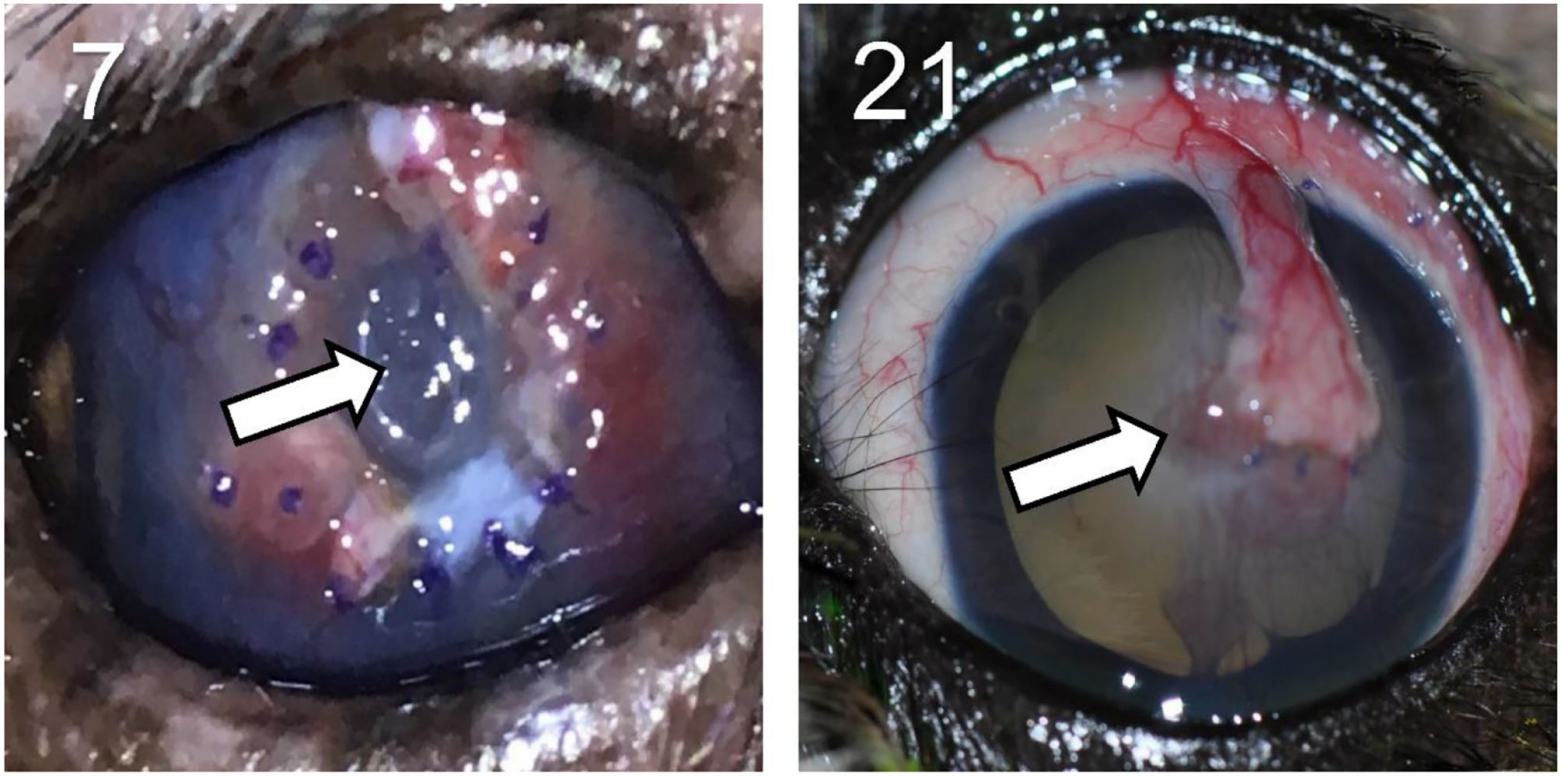
Clinical photographs showing disintegration (left) and focal dehiscence (right) of the conjunctival pedicle flap surgically positioned at a previous visit in canine eyes with culture-confirmed bacterial keratitis. Patients ID are shown in the top left (see Table 1 for additional details).

In the other 28/30 cases (93.3%), the bacteria isolated from the corneal ulcer was *susceptible* to at least one of the topical antibiotics used at the initial visit; however, the clinical outcome was not always favorable despite aggressive medical management (e.g., antibiotics applied topically up to 12 × daily) combined with corneal grafting procedures in selected cases. In cases managed with medical therapy alone (22/28, 79%), 15 eyes healed uneventfully within 7–30 days (example case #20—Figure 3), 1 eye (case #2) initially worsened then healed after the addition of a new antibiotic, 3 eyes (#6, #13, and #16) deteriorated and required enucleation due to discomfort and vision impairment, and 3 eyes (#4, #17, and #27) required surgical stabilization with a conjunctival pedicle flap within 3–14 days (example case #27—Figure 3). In cases managed with medical therapy and surgical stabilization at the first visit (6/28, 21%), only 3 healed uneventfully (cases #9, # 14, and #29) while the other 3 patients (cases #7, #8, and #21) experienced localized suture/graft dehiscence presumably due to ongoing keratomalacia underneath the graft (Figure 4).

## Discussion

Bacterial keratitis is a vision- and globe-threatening condition that requires prompt diagnosis and treatment to prevent unfavorable outcomes. Timely antimicrobial therapy must be started on the basis of clinical and laboratory evaluation; however, clinical success can be hindered by several challenges such as the rise of antimicrobial resistance (6, 20–22) or progression of keratomalacia despite the appropriate use of antibiotics (23–25). Here, we describe an important challenge not previously recognized in the scientific literature, that is, the negative impact of protein binding in tear fluid on the efficacy of antibiotics commonly used to treat ocular infections. The present work was conducted in dogs, a species that represents a robust and translational large animal model for comparative ophthalmology research given similarities in ocular anatomy (9), physiologic parameters pertinent to topical route of drug administration (18, 26), and prevalence of common micro-organisms responsible for infectious keratitis between humans and dogs (6, 23, 27, 28).

The reduction in antibacterial activity from protein binding is well-recognized in plasma and other infection sites that contain high levels of proteins (29). For instance, the albumin-rich composition of skin exudates is known to decrease the antibacterial activity of common antiseptics used to treat skin wounds in humans (30, 31). In fact, the binding of antimicrobials to proteins has two important consequences from a chemotherapeutic point of view. First, the protein-bound drug is essentially without antimicrobial activity as the drug is no longer able to collide with the microbes (32). Second, the bound drug is not diffusible as large proteins cannot permeate through semipermeable membranes (33). Unfortunately, there is currently no standardization or requirement for *in vitro* work to account for the impact of protein binding on antimicrobial activity, notably for antibiotic susceptibility testing using the standard medium.

Mueller-Hinton broth is the standard liquid medium recommended by the Clinical and Laboratory Standards Institute for susceptibility testing of most aerobic and facultative anaerobic bacteria (1, 19). MHB provides optimum conditions for bacterial growth and more closely resembles plasma and serum in terms of pH, osmolality and electrolytes composition (Na+, K+, Cl–) than other media (29). To address the lack of proteins in MHB and better mimic *in vivo* conditions at the infection site, two main approaches are commonly described in the literature: (i) Incorporate serum to the test medium, ensuring bacterial growth is not inhibited by the adjusted medium since serum contains antimicrobial-acting substances (34), or (ii) Incorporate albumin alone to the test medium as this protein represents the major actor in drug binding (29, 34). Both strategies were employed in the present work, adjusting the study design in order to be clinically relevant to the field of ophthalmology. First, albumin levels were not empirically set to 4% (~levels in human serum) as described elsewhere (29); rather, albumin levels purposely represented a wide range of concentrations detected in tear fluid of dogs with ocular disease, based on previous canine reports (8, 10) as well as prospective evaluation of canine patients diagnosed with ulcerative keratitis at the authors' institution. Second, the authors investigated actual tear fluid (instead of serum or plasma) by collecting sufficient lacrimal volume with a recently described technique (ophthalmic sponges) (16), assessing whether microbiological results would differ between tear fluid and albumin-only solutions. In fact, tears represent a complex biological fluid that contain many antimicrobial compounds (e.g., lysozyme, lactoferrin, immunoglobulin A) (35) as well as proteins other than albumin that could theoretically affect the bioavailability of drugs (e.g., alpha-1-acid glycoprotein) (13). In both experiments (albumin solutions, tear fluid), it is important to note that the authors confirmed the lack of antimicrobial activity from the adjusted test media alone (>2,400 positive controls).

Albumin concentrations had a significant impact on the MIC of several antibiotics commonly used in ophthalmology, a finding confirmed for 3 bacterial species (*Staphylococcus* sp., *Streptococcus* sp., *Pseudomonas* sp.) that are commonly identified in ocular infections of canine (6, 28) and human patients (23, 27). The impact of protein binding may be overlooked in cases of highly susceptible bacteria (34), therefore 10 different isolates were tested for each bacterial species making the present findings more broadly applicable.

Similar to studies on protein binding in blood, the present study found that the impact of protein on MIC depended on several factors including (i) albumin concentration, (ii) specific antibiotic, and (iii) bacterial species (12, 33, 36). (i) As expected, changes in MIC were more frequent and more pronounced as albumin concentrations increased in the test medium. (ii) Selected antibiotics (e.g., amikacin, cefazolin) were not affected by the addition of albumin at all, regardless of the protein concentration, as previously reported for fosfomycin and moxifloxacin (37); in contrast, antibiotics such as ofloxacin were much more “susceptible” to the impact of albumin binding on MIC, although the underlying reason is unclear and requires further investigation. (iii) Bacterial species were unevenly affected by albumin addition to the test medium, as exemplified by differences in MIC changes of chloramphenicol for *Staphylococcus* sp. (100% isolates) and *Streptococcus* sp. (0% isolates) despite known susceptibility of both bacterial species to this broad-spectrum antibiotic (6).

Unlike studies on protein binding in blood (38), the MICs did not increase proportionally to the degree of protein binding inherent to each antibiotic. This discrepancy could be explained by differences in protein and drug concentrations in blood vs. tear fluid. Compared to blood, albumin concentrations are ~10–100-fold lower in tears (8, 10, 39) while drug concentrations are relatively higher in tears, especially after topical drug administration (26, 40). Thus, albumin levels used in the present study (i.e., purposely selected to be relevant for the ocular surface) are likely to get saturated by the antibiotic molecules, rendering any excess drug to become unbound regardless of the drug's protein binding properties (33). Another potential explanation is related to the protein binding values selected for data analysis. Binding values were mostly derived from human data (due the lack of comprehensive characterization in dogs) despite known species differences (36, 41), and values were extrapolated from the literature instead of testing the actual free fraction of each antibiotic in the test medium (42). Such discrepancies could help explain the negative association that was detected between percent protein binding and percent changes in clinical interpretation.

A secondary objective of the study was to assess the impact of actual tear fluid on MICs of ophthalmic antibiotics, collecting tears from canine patients with ocular surface inflammation. We originally hypothesized that MIC changes would be greater with tear fluid vs. albumin solutions due to the presence in tears of proteins other than albumin that could bind to drugs, as well as compounds such as fatty acids (9) that could enhance the affinity of albumin to drugs (34). However, no significant differences were noted in MICs of antibiotics exposed to canine albumin solution vs. canine tear fluid containing the same level of albumin. It is likely that albumin represents the predominant protein that binds to drugs in tear fluid, similar to plasma (12), and that other tear proteins (e.g., lactoferrin, lysozyme) only have a minimal and non-clinically significant impact on drug bioavailability. The tear fluid experiment yielded two important findings: (i) albumin solution compounded from lyophilized powder is of good quality and mimics the effects of albumin naturally occurring in biological fluids *in vivo*, as previously shown in another canine study (43); (ii) supplementation of test medium (MHB) with albumin is sufficient for antimicrobial testing of ocular surface infections.

MIC changes from drug-protein binding in tear film may have serious repercussions for clinical patients. First, with higher MIC values, bacterial isolates are exposed to sub-therapeutic levels of antibiotics for longer durations, at risk of promoting the development of antibiotic resistance (44) that can become problematic for the general population. Looking at second-generation fluoroquinolones for instance, we found that MIC changes were much more common for ofloxacin (87% of all bacterial isolates) than ciprofloxacin (3%), and this finding may help explain the higher rate of resistance to ofloxacin vs. ciprofloxacin that is reported in selected studies (6, 45). Second, a close relationship exists between MICs and clinical outcomes for the individual patient. In a large sample (*n* = 391 eyes) of human patients with bacterial keratitis treated with ciprofloxacin, Wilhelmus et al. reported a significantly lower rate of clinical improvement (by 43%) and clinical cure (by 29%) among corneal infections having ciprofloxacin MICs above 1.0 μg/mL compared with those with more sensitive isolates (46). Further, a significant correlation was detected between MIC and the size of corneal infiltrate/scar in one study (47), and between MIC and overall clinical outcome for most bacteria (except for coagulase-negative staphylococci and *Streptococcus* sp.) in another study (23).

Unfavorable clinical outcomes are also reported in veterinary patients with bacterial keratitis. Despite intensive medical management, worsening of corneal disease was noted in 30% of eyes in the present study, 30% in a report by Pot et al. (24) and 46% in a recent publication (25). In fact, clinical deterioration was serious enough in 10% of our canine patients (cases #6, #13, and #16) that it required surgical removal of the eye due to loss of vision and serious discomfort. Undoubtedly, optimizing *in vitro* testing to better mimic *in vivo* conditions could improve these clinical outcomes by assisting clinicians to select the most appropriate antibiotic(s). Following the addition of albumin to the test medium at clinically relevant concentrations (0.1%), the extrapolated clinical interpretation of ofloxacin switched from *susceptible* to *intermediate* in 5 canine patients (cases #2, #4, #7, #17, and #21), all of whom experienced complications with corneal healing (e.g., progression of corneal ulcer, graft dehiscence). Bacterial keratitis worsened in another patient (case #27) in whom the clinical interpretation for gentamicin was presumably *susceptible* but became *resistant* following the addition of 0.1% albumin to the test medium. On the other hand, it must be emphasized that changes to MIC or clinical interpretation do not ineluctably result in clinical deterioration, as exemplified by case #28 (proper healing despite tobramycin switching from *intermediate* to *resistant*) and the lack of correlation between MIC and clinical outcome for selected bacterial species (23). In fact, antibiotic susceptibility is only one of many factors associated with outcome in bacterial keratitis, explaining up to 13% of the variance in outcome according to one study (48); other predictive factors include ulcer's characteristics (size, depth), microbial virulence, compliance with medications, and absence/presence of concurrent systemic or ocular diseases (e.g., diabetes mellitus, keratoconjunctivitis sicca) (24, 25, 49).

The main limitation of the study is related to the static nature of the experiment. We aimed to provide an approach that would be readily feasible for microbiologist while better mimicking *in vivo* conditions of patients with bacterial keratitis—assessing common bacterial species and clinically relevant levels of albumin—yet even this optimized *in vitro* AST cannot capture the complex dynamics of the ocular surface. Contact time between the antibiotic and albumin is much longer in the *in vitro* setting (16–24 h incubation) than on the ocular surface (<30 min) (9). Topically administered medications are rapidly lost due to reflex blinking and efficient nasolacrimal drainage (18, 26), with only ~55 and 5% of drug remaining in the tear film at 1 and 30 min following eyedrop administration (40). As such, reversibility in drug binding (i.e., bound drug becomes unbound and vice versa) (34, 50) is likely to occur *in vitro* but is either absent or limited in the tear film. In other words, the fraction of an antibiotic that is bound to albumin in tears can be considered “wasted” from a pharmacological standpoint, washed off from the ocular surface before bound-to-unbound transition can allow the drug to exert its antimicrobial activity. Another study limitation is the assumption that protein binding values reported in the literature (e.g., 50% for moxifloxacin) would reflect the binding conditions in the actual test system (i.e., AST plates). This assumption may be misleading and it is therefore suggested to measure the actual free (unbound) antibiotic in the *in vitro* setting (32, 42). Last, the study's focus on MICs as the main outcome has inherent drawbacks. MIC testing is easy to perform and is the most widely used parameters for pharmacokinetic-pharmacodynamic modeling of antibiotics (29, 51). However, small changes in MIC values might be overlooked as the method uses only 2-fold dilution steps; further, MIC testing detects only visible growth (i.e., 100-fold increase in bacterial count after incubation) and is therefore unable to distinguish between less pronounced bacterial growth and bacterial killing. As such, the MIC method may be unsuitable for investigating antibiotics that display low or moderate protein binding (≤ 50%). For instance, the impact of protein binding on moxifloxacin was overlooked with the MIC approach but the impact was clear when time-killing curves were used (37).

Strategies to minimize drug-protein interactions and enhance ocular bioavailability were discussed in a recent publication by Sebbag et al. (43), including the use of higher drug concentration (to compensate for the fraction lost to albumin binding in tears), stabilization of the blood-tear barrier (to reduce albumin leakage from plasma to tears), or competitive inhibition of protein binding (e.g., cetylpyridinium chloride). Specific to patients with bacterial keratitis, clinicians can consider fortified antibiotics instead of commercially available concentrations (e.g., 1.4% instead of 0.3% gentamicin) (49), frequent eyedrop instillation to saturate the tear film with antibiotics (e.g., loading dose every 5–15 min then hourly thereafter) (49), as well as non-antibiotic strategies such as corneal collagen cross-linking (24, 49).

In conclusion, tear levels of albumin ≥0.05% impacted the efficacy of selected ophthalmic antibiotics as only the unbound portion of an antibiotic is microbiologically active (12, 13, 32). The present findings could improve decision making of clinicians managing bacterial keratitis, reduce development of antimicrobial resistance, influence current guidelines set by CLSI, and serve as a reference for bacteriological evaluations across medical fields and across species.

## Data Availability Statement

The datasets generated for this study are available on request to the corresponding author.

## Ethics Statement

The animal study was reviewed and approved by the Institutional Animal Care and Use Committee at Iowa State University.

## Author Contributions

LS conceptualized and designed the study in consultation with DK and JM. LS, VB, DK, and AP performed the experiments. LS and JM analyzed the data. All authors wrote the manuscript.

## Conflict of Interest

The authors declare that the research was conducted in the absence of any commercial or financial relationships that could be construed as a potential conflict of interest.

## Publisher's Note

All claims expressed in this article are solely those of the authors and do not necessarily represent those of their affiliated organizations, or those of the publisher, the editors and the reviewers. Any product that may be evaluated in this article, or claim that may be made by its manufacturer, is not guaranteed or endorsed by the publisher.
