# Supplementary material for: Albumin in Tears Modulates Bacterial Susceptibility to Topical Antibiotics in Ophthalmology
Source: Front Med (Lausanne). 2021 Nov 30;8:663212. doi: 10.3389/fmed.2021.663212 (PMC8669104; doi:10.3389/fmed.2021.663212)

Supplementary Material

Figure 1. Six assay plates were used for each of the 30 bacterial isolates. One blank plate served as positive control, confirming the lack of antibacterial activity from albumin alone following the addition of the protein (0-2%) to the bacterial broth. Five Sensititre™ JOEYE2 plates assessed the MICs of 17 ophthalmic antibiotics in the absence (0%) or presence (0.01-2%) albumin in the bacterial broth.

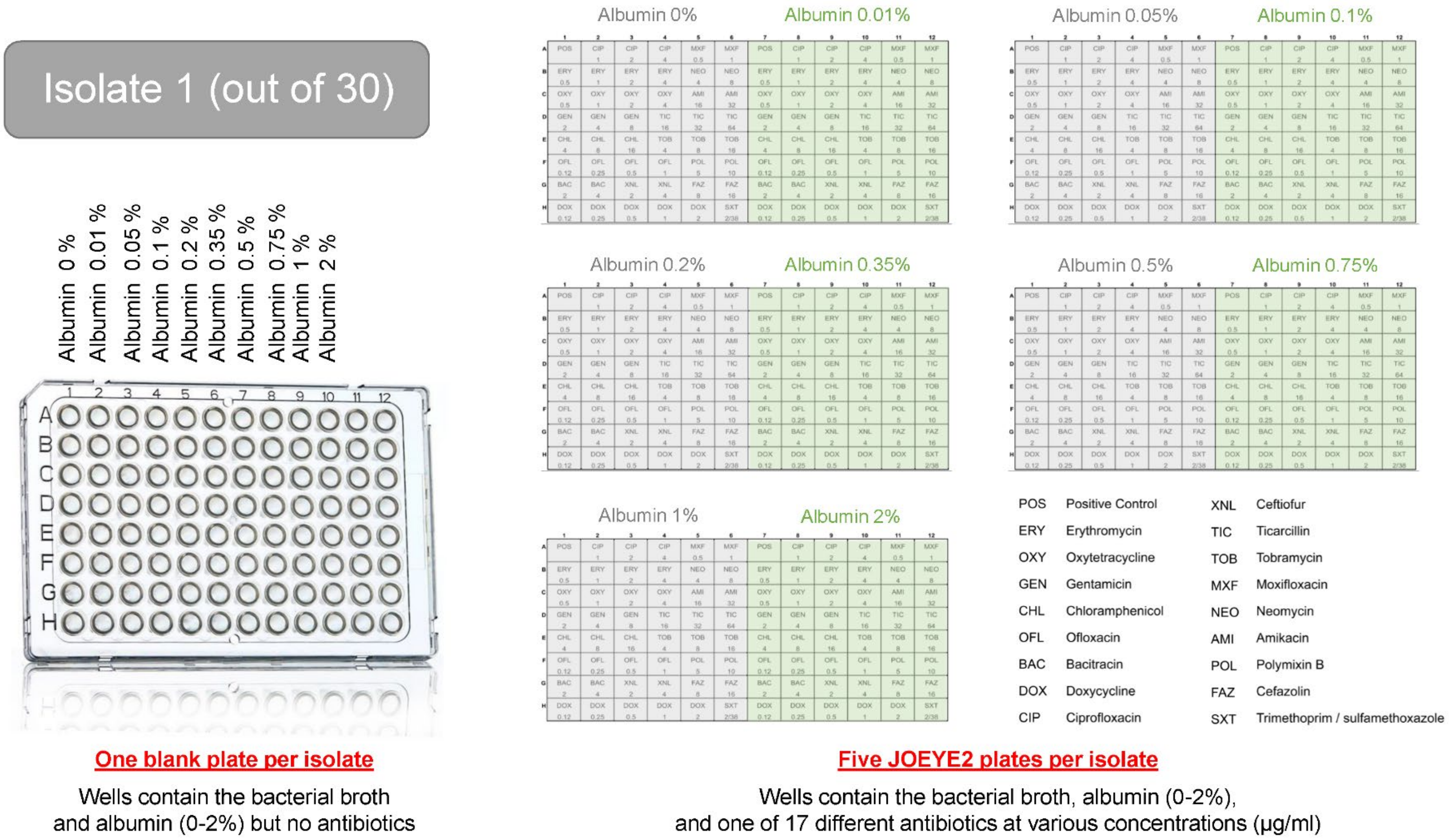

Figure 2. Positive bacterial growth noted in all wells of a blank plate containing bacterial broth (*Pseudomonas aeruginosa* isolate) and canine albumin: 0% (column 1), 0.01% (column 2), 0.05% (column 3), 0.1% (column 4), 0.2% (column 5), 0.35% (column 6), 0.5% (column 7), 0.75% (column 8), 1% (column 9) and 2% (column 10).

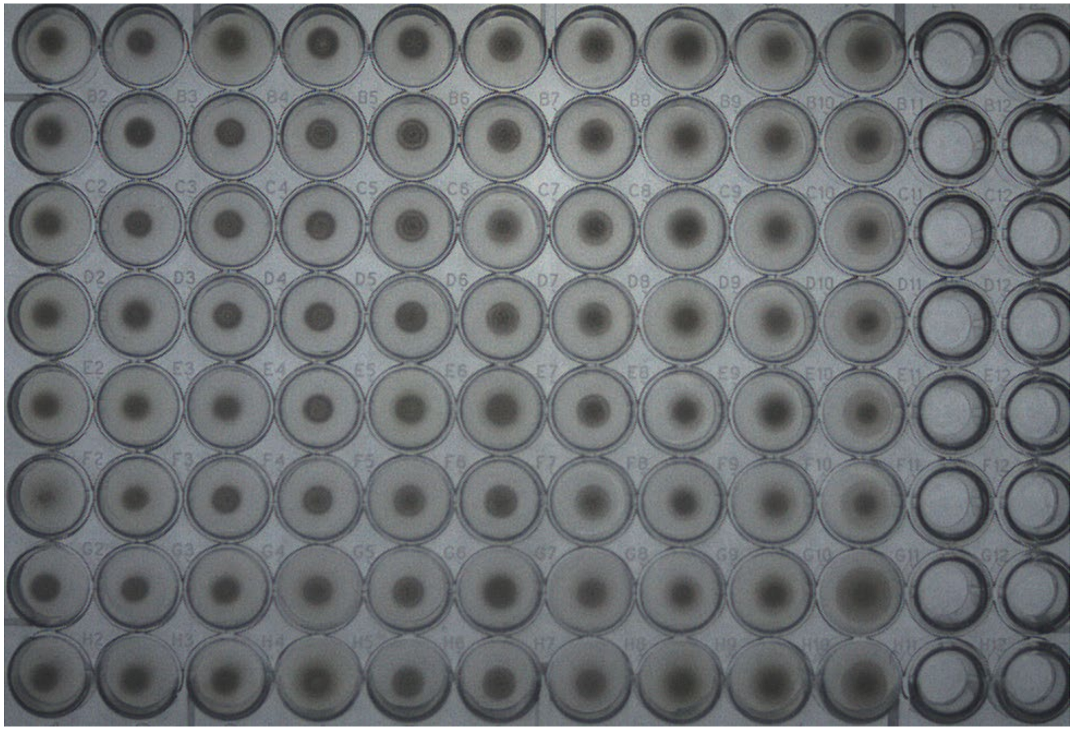

Supplement: Supplementary file 1 [file Data_Sheet_1.PDF]
